# A Novel User Classification Method for Femtocell Network by Using Affinity Propagation Algorithm and Artificial Neural Network

**DOI:** 10.1155/2014/253787

**Published:** 2014-07-16

**Authors:** Afaz Uddin Ahmed, Mohammad Tariqul Islam, Mahamod Ismail, Salehin Kibria, Haslina Arshad

**Affiliations:** ^1^Space Science Centre (ANGKASA), Universiti Kebangsaan Malaysia (UKM), 43600 Bangi, Selangor, Malaysia; ^2^Department of Electrical, Electronic and Systems Engineering, Universiti Kebangsaan Malaysia (UKM), 43600 Bangi, Selangor, Malaysia; ^3^Centre of Artificial Intelligence Technology, Faculty of Information Science and Technology, Universiti Kebangsaan Malaysia (UKM), 43600 Bangi, Selangor, Malaysia

## Abstract

An artificial neural network (ANN) and affinity propagation (AP) algorithm based user categorization technique is presented. The proposed algorithm is designed for closed access femtocell network. ANN is used for user classification process and AP algorithm is used to optimize the ANN training process. AP selects the best possible training samples for faster ANN training cycle. The users are distinguished by using the difference of received signal strength in a multielement femtocell device. A previously developed directive microstrip antenna is used to configure the femtocell device. Simulation results show that, for a particular house pattern, the categorization technique without AP algorithm takes 5 indoor users and 10 outdoor users to attain an error-free operation. While integrating AP algorithm with ANN, the system takes 60% less training samples reducing the training time up to 50%. This procedure makes the femtocell more effective for closed access operation.

## 1. Introduction

The number of cellular users has increased significantly over the last few decades. To some extent, the cellular operators were able to provide the increasing demand of voice and data services. However, due to massive growth of multimedia applications, the demand has reached beyond the limit where the existing macrocell cannot support such high node density. The increasing number of indoor data traffic (more than 70%) has made it quite difficult for the operators to provide quality coverage using the existing macrocell [1]. An alternative to this is that femtocell opens up a cost-effective solution by offloading excess voice and data traffic. It provides high quality indoor coverage which connects to the core network through wired backhaul [2]. Without proper planning, vast deployment of femtocell causes interference problem in dense heterogeneous network. Overlapping of coverage zones of both macrocell and femtocell is mostly subjected to unwanted handover, cell overshooting, and high mobility events. On the contrary, the users buy the femtocell to enjoy the service of high quality indoor coverage [3–5]. Access control algorithm is introduced to macrofemtocell network to minimize the interference caused by excess mobility event. Among the three access control mechanisms, closed access allows only particular users (mainly indoor users) to get access to the network [6–9]. In such technique, the outdoor users cannot get access to the femtocell and the mobility event reduces. However, under supreme coverage of femtocell, the outdoor user attempts to change base station that creates signaling congestions in the network. Therefore, multielement antenna configuration for femtocell application has been proposed in various articles. It utilizes beam-forming technique to control the coverage of femtocell [10–13]. The antennas are usually mounted on the vertical surface of the device with an individual scanning angle and separation distance. It creates null coverage in the interference regions and optimizes the coverage to avoid supreme outdoor coverage. Thus far, all the efforts aimed to reduce the interference by making null coverage in the affected region.

Smart antenna concept is an add-on to wireless network in recent years. Direction of arrival (DOA) estimation and beam steering are considered the fundamental function of the smart antenna [14–16]. In addition, new features, like user localization based on distinctive characters of users' signals, are also under consideration. Array antennas give flexibility to identify the users in an adaptive spatially sensitive manner. It represents leading-edge smart antenna approach by using diverse signal-processing algorithm adjusted to real time. In this paper, a novel technique for user classification is proposed for multielement femtocell device by using artificial neural network (ANN). Clustering algorithm of affinity propagation (AP) is also introduced to make the process faster and effective. In multielement femtocell, each of the antennas has different receiving gain in different angle that gives a set of received power pattern for every user. Based on this, the femtocell is trained to identify the indoor and outdoor users. To model the nonlinear relationship between the indoor and outdoor user, ANN is trained using randomly generated user samples. The trained ANN allows the femtocell to select the indoor and outdoor users from the antenna end. In addition, the training process is upgraded using AP clustering algorithm. This paper focuses on unwanted user admission control in femtocell to decrease the unwanted handover and signaling congestion. As femtocell distinguishes between the users after a certain time, it does not accept users outside the house, which results in a less number of handover requests. The performance of the proposed technique is shown as percentage of error rate in identifying the correct users. The remainder of the paper is described as follows; user categorization technique is explained in Section 2 and detailed structure of the ANN and AP clustering algorithm is described in Sections 2.1 and 2.2, respectively. Results and Discussions are in Section 3 and Conclusion is in Section 4.

## 2. User Categorization in Closed Access Femtocell

Closed access mechanism in femtocell network avoids unwanted handover and mobility events in dense macrofemtonetwork. The users are predefined and femtocell only allows access to particular group of users. In case of superior coverage, which is beyond the threshold limit of the received signal level, outdoor users want to switch serving cell. As a result, the femtocell gets continuous handover request on SDCCH (stand-alone dedicated control channel) from the outdoor user. This induces signalling congestion that encompasses the core network for each request. Most of the time, this event occurs due to overshooting of the femtocell in unwanted direction. Use of multielement antenna instead of omnidirectional antenna optimizes the coverage of femtocell and minimizes the overshooting effect. However, in initial stage, femtocell does not have any prior knowledge of house's dimension and its own position. In such condition, multielement antenna also creates the overshooting problem. In multielement femtocell device, the antennas are faced in different direction, which allows forming of directional beam for particular user to avoid interference. Since all the proposed multiantenna concepts used planner antennas like PIFA (planner inverted-F antenna) and patch antenna, previously designed microstrip antenna has been used in this paper to simulate the femtocell device. The antenna was designed for LTE band 7 [17]. It has a directional gain pattern that gives different receiving gain for different position of the user. A 6-element antenna structure is considered for the femtocell device with a scanning angle of 60° degree each. For a particular user in the uplink, the femtocell will have 6 different received power patterns. The relation between the received power and antenna gain, which was shown in Friis transmission equation, is given below [18]:

(1) $$\begin{array}{c} P_{r}=P_{t}\times G_{t}\left(\theta_{t},\varphi_{t}\right)\times G_{r}\left(\theta_{r},\varphi_{r}\right)\times {\left(\frac{\lambda }{4\pi R}\right)}^{2}, \end{array}$$

(2) Pr(dBm)=Pt(dBm)+Gt(θt,φt)(dB)+Gr(θr,φr)(dB)+20 log10(λ4πR)︸free  space  pathloss(dB),

where *P*_*r*_ and *P*_*t*_ are the receive and transmit power, respectively. *G*_*t*_(*θ*_*t*_, *φ*_*t*_) and *G*_*r*_(*θ*_*r*_, *φ*_*r*_) are the transmit and receive antenna gain at the receiver and transmitter direction, respectively.

**Figure 1:**
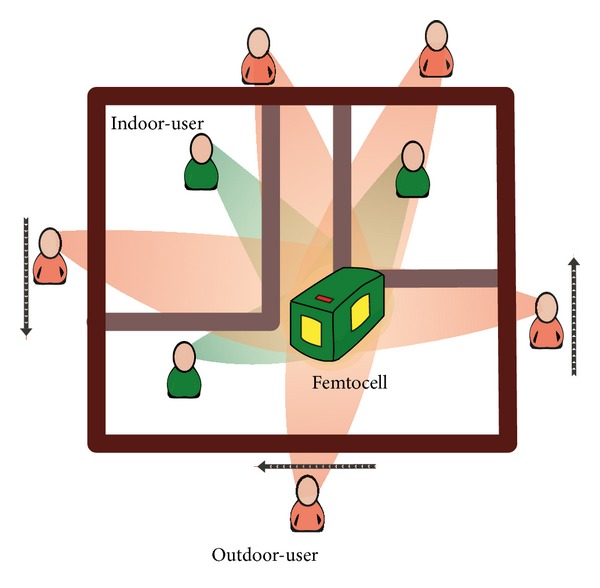
User pattern in closed access femtocell network.

The transmitting antenna of the user's equipment is assumed to be omnidirectional. Even if the antennas are directional, the received signal strength on the antenna patch will change scantly as the mutual distance among the antennas is very small compared to the distance from the femtocell to the user equipment. In (2), the receiving gain and free space path-loss for every user are different. Comparing with the distance between the users and femtocell, the size of the femtocell is quite small. As a result, the free space path-loss is almost the same for each antenna element.  Figure 1 visualizes the scenario of the above discussion.

Femtocell antennas respond to an incoming wave from a given direction according to the pattern value in that direction. Each of 6 antenna elements holds different gain pattern in each direction. Therefore, the received power varies due to the prospective antenna gain. The variation of received power is used to differentiate between the outdoor and indoor users. Femtocell performs mapping from incident wave to the received power pattern. The neural network is trained to do the inverse mapping. It uses the vectors comprised of energy, *E*, from all antennas over multiple instances of *n*:

(3) $$\begin{array}{c} E_{i,n}=\overset{T(n+1)}{\underset{n\times T}{\int }}P_{i}\left(t\right)dt;\;i=1,2,3,4,5,6,\\ \bar{E}=\left[\begin{array}{c} E_{1,n}\\ \vdots \\ E_{6,n} \end{array}\right], \end{array}$$

where* T* is the sampling period and *n* = 0,1, 2,3….

In the training stage, the ANN learns the behaviour of indoor and outdoor users using the value of $\overset{-}{E}$. The network categorizes the user based on the previous learning. For the task, a simulated environment is developed in MATLAB. Indoor and outdoor users are randomly generated using uniformly distributed pseudorandom number. A 2D layout of a house is also designed considering the indoor and outdoor walls. Moreover, AP clustering algorithm is used to filter out the best possible samples from randomly generated data points. It allows the ANN to learn faster with the same level of accuracy but a less number of iterations. After the training, another set of random samples are generated to evaluate the performance of the network. Standard path-loss model and additive white Gaussian noise are considered in free space path-loss calculation:

(4) Pathlossf(db)=38.46+20 log10D+0.7d2D,indoor +18.3n((n−2)/(n+1)  −0.46)+wLiw,

where *D*, *w*, *n*, 0.7*d*_2*D*,indoor_, and Pathloss_*f*_ are distance, number of walls, number of floors, penetration loss inside the house, and path-loss of the users, respectively [19].

**Figure 2:**
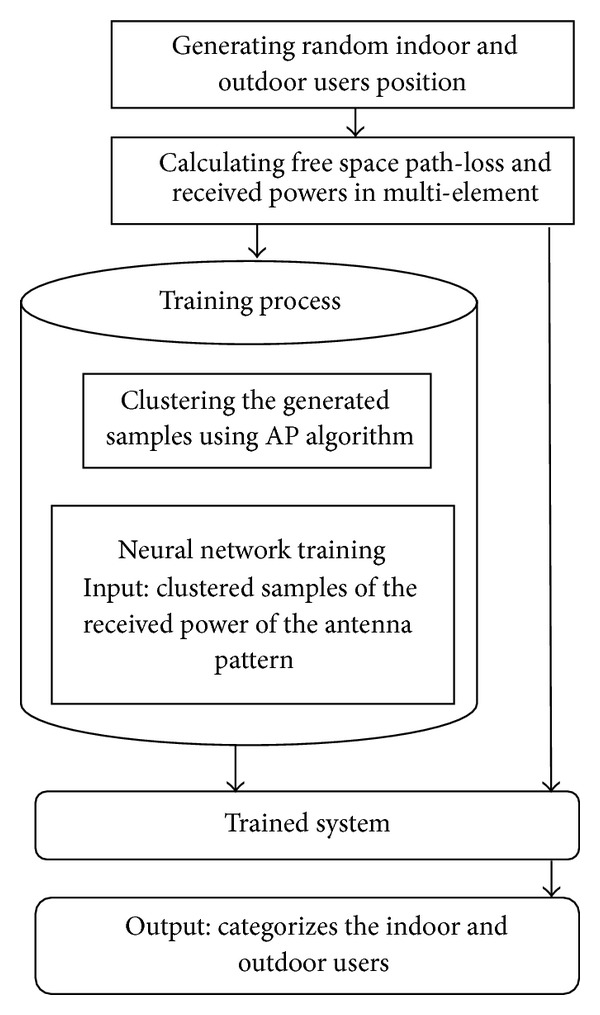
Proposed femtocell user selection technique using ANN and AP algorithm.

Using AP algorithm and ANN, femtocell determines the users' category to allow access. For random values of $\overset{-}{E}$, the neural network determines the users' category by giving an output of “+1” or “−1.” The details of process is projected in a flow chart in Figure 2.

### 2.1. Artificial Neural Network for User Categorization

Artificial neural network (ANN) is a machine-learning process that is modelled after the brain architecture. Like the brain's smallest cell neuron, it contains hundreds of processing units wired together as a complex network. It is trained using the sample data to predict the behaviour of the future data [20]. User categorizing is a supervised learning process. A model is prepared through a training process where it is required to make predictions and is corrected when those predictions are wrong. The training process continues until the model achieves a desired level of accuracy on the training data. In general, algorithms are presented in groups by similarities in terms of their operation process and function. There are algorithms that could easily fit into multiple categories like learning vector quantization. It is both an instance-based method and a neural network inspired method. There are categories that have the same name that describes the problem and the class of algorithm such as regression and clustering. The popular machine leaning algorithms are regression, instance-based methods, regularization methods, decision tree learning, Bayesian, kernel methods, clustering methods, association rule learning, deep learning, dimensionality reduction, ensemble methods, and artificial neural network [21]. However, in machine learning algorithms themselves, there is no perfect model, just a good enough model depending on how the application layout is designed. ANN has many attractive theoretic properties, specifically, the ability to detect nonpredefined relations such as nonlinear effects and/or interactions. These theoretic advantages come at the cost of reduced interpretability of the model output. Many authors have analysed the same data set, based on these factors, with both standard statistical methods and ANN [22–24].

In the proposed technique, multilayer perceptron feed forward backpropagation (MLPFFBP) neural network is used to categorize the users. MLPFFBP uses error backpropagation to adjust the weights of the neurons. There are two passes in the layers of the network: forward pass and backward pass. The network consists of three layers: input layer, output layer, and the hidden layer. The input layer is fed with initial data. The output layer gives the desired solution. In between there exists a series of hidden layers. The primary layer is connected with the input layer and the last layer is connected to the output layer. Each subsequent layer is connected with the previous layer. Based on the network design, each hidden layer consists of multiple numbers of neurons. The neurons use differentiable transfer function to generate the output. During the training period, the input and output values of the network are specified and based on these values and the hidden layer builds up a set of weights for the neurons [25].

The differentiable transfer function (*tansig*) used here is a sigmoid function. In multilayer sigmoid function, if the input vector is very large, the weight becomes so small to prevent the transfer function being saturated. Thus, the gradient will be very small and the neural network will be very slow. On the contrary, higher number of training samples with higher number of neurons makes the network more accurate but such a process makes the network bulky and time-consuming. For this, preprocessing steps are added in-between the input layers and the hidden layers. The performance of the neural network is made more effective by using a preprocessing step in training sample selection. In this case, AP clustering algorithm is used to select the best-suited samples for the network training.

**Figure 3:**
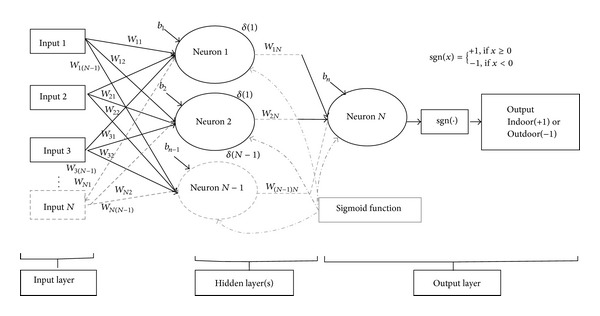
Structure of MLPFFBP in the proposed technique.

In Figure 3, *b*_1_, *b*_2_,…, *b*_*n*−1_, *b*_*n*_ and *w*_11_, *w*_12_ ⋯ *w*_21_, *w*_22_ ⋯ *w*_*N*(*N*−1)_, *w*_*NN*_ are the biases and the weights of the network nodes, respectively. Biases are also considered the primary weights that are initially put as 1. Moreover, “signum” function is used to compute the actual response of the perceptron. The final output from the last neuron passes through the “signum” function that gives the binary output.

The transfer function is

(5) $$\begin{array}{c} \varphi \left(v\right)=\frac{1}{1+\exp\left(-v\right)}. \end{array}$$

The signum function is

(6) $$\begin{array}{c} \mathrm{sgn}\left(x\right)=\{\begin{array}{cc} +1, & \text{if}\;x\geq 0\\ -1, & \text{if}\;x<0. \end{array} \end{array}$$

The weights are calculated as

(7) $$\begin{array}{c} w\left(n+1\right)=w\left(n\right)+\alpha *w\left(n-1\right)+\eta *\delta \left(n\right)*y,\\ \delta \left(n\right)=\varphi^{\prime }\left(v\right)*\left(d-y\right), \end{array}$$

where *α*, *η*, *d*, *y*, and *δ* are the mobility factor, the training parameter, the desired output, the real output, and the local gradient for the nodes of the network, respectively [26, 27]. After the training process of the network, the femtocell takes the 6-element antennas received power as input and gives the category of the users in the output.

### 2.2. Affinity Propagation Algorithm for Selecting the Best Samples

AP algorithm is a recent clustering algorithm proposed by Frey and Dueck [28]. It is widely accepted because of its high quality set of cluster samples. The proposed user classification in neural network is a supervised technique. The performance of the network is subjected to the nature and quantity of the training samples. Higher number of training samples led to precise values of the neurons' weight, but it makes the training process slower. Clustering of data set based on similarities is a vital step in data analysis problem. A common practice in both supervised and unsupervised learning is to cluster the data based on the similarities [29, 30]. Affinity propagation (AP) is the latest clustering algorithm that reduces the redundancy of the training data set. It accelerates the computing process of ANN by reducing the sample numbers.

Traditional clustering algorithms follow random selection of initial data subset as exemplars and refine it iteratively. AP takes an input set of pairwise similarities between the data points and finds the clusters based on maximum total similarities between the exemplars and the data points [31]. The real messages are exchanged between the data points until the finest set of exemplars and corresponding clusters progressively emerges. It has a better clustering performance than* K*-means,* K*-medians, fuzzy *c*-means, Hill combining (HC), and self-organizing map (SOM) algorithms [32, 33]. It is computationally efficient and simple to implement and customize. In AP algorithm, all the sample data points are considered a possible candidate to be the desired exemplars. Each step exchanges real-valued messages between them until a superior set of exemplar shows up. Messages are updated based on simple formulae that reflect on the sum-product or max-product. It updates the rules until the magnitude of the messages reflects on the current affinity for choosing another data point as its exemplar. Each data point is considered a note in the network. The process of the algorithm is described briefly below.

Input is a set of pairwise similarities as

(8) {s(i,k)}=−||xi−xk||2,i≠k(squared  Euclidean  distance),where,  (i,k)∈{1,…,N}2,  s(i,k)∈R.

Here *s*(*i*, *k*) ∈ *R* indicates how well suited the data point *k* is as an exemplar for data point* i*.

For each data point *k*, a real number *s*(*k*, *k*) represents the preference that is to be considered as an exemplar:

(9) $$\begin{array}{c} s\left(k,k\right)=\rho ,\;\forall k\in \left\{1,\ldots ,N\right\}. \end{array}$$

Initialization: set availabilities to zero,  for all *i*, *k* : *a*(*i*, *k*) = 0.

Repeat responsibility and availability updates until convergence:

(10) ∀i,k:r(i,k)=s(i,k)−max⁡⁡[s(i,k′)+a(i,k′)],∀i,k:a(i,k) ={∑max⁡[0,r(i′,k)], for  k=imin⁡⁡[0,r(k,k)] +∑i′:i′∉{i,k}max⁡⁡[0,r(i′,k)], for  k≠i.

Output is assignments $\hat{c}=({\hat{c}}_{1},\ldots ,{\hat{c}}_{N})$, where ${\hat{c}}_{i}=\arg\;\text{ma}{\text{x}}_{k}[a(i,k)+r(i,k)]$. Here ${\hat{c}}_{i}$ indexes the cluster's exemplar at which point *i* is assigned. If point *i* is a cluster with point *k* as the exemplar, then ${\hat{c}}_{i}=k$ and ${\hat{c}}_{k}=k$ [34].

## 3. Results and Discussions

**Figure 4:**
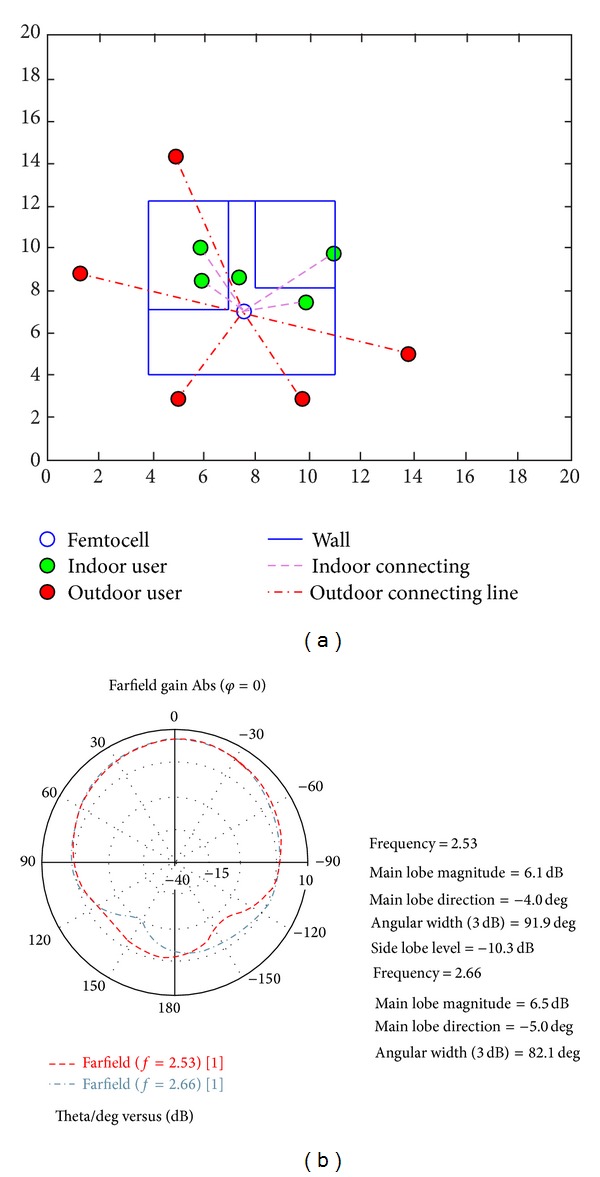
(a) Layout of the simulation environment. (b) Radiation pattern of the microstrip antenna at 2.53 GHz and 2.66 GHz.

A layout of functioning area is modelled with a femtocell in the middle of the house. Six-microstrip antennas are operating with 60° separation angle on the same axis inside the femtocell device. A previously designed microstrip antenna is used here to configure the directive gain pattern of each antenna element [17]. The house has indoor and outdoor walls that decrease the strength of the signal based on their thickness. Initially random indoor and outdoor users are generated and the received powers are measured. The dimension of the house is set to 7 m × 6 m. In Figure 4(a) the users and the house are plotted in a 20 m × 20 m window. The radiation pattern of the microstrip antenna is shown in Figure 4(b).

To demonstrate the performance of the technique, ANN is initially trained without using AP clustering. Random samples are generated by varying the numbers of indoor and outdoor users. In the performance analysis stage, again, random samples are generated to categorize users using the previous experiences. The system parameters that have been used in the simulation are given in Table 1. In the model, the outdoor wall loss is considered higher than the indoor wall. One of the reasons is that usually the outdoor walls are thicker than the indoor wall with more concrete and steel materials for the foundation or shape. This increases the loss exponent of the outdoor walls. Another reason is that outdoor walls are more subjected to rust and moist from the environment that weakens the incoming signal [35].

### 3.1. Femtocell Network Performance with ANN

**Figure 5:**
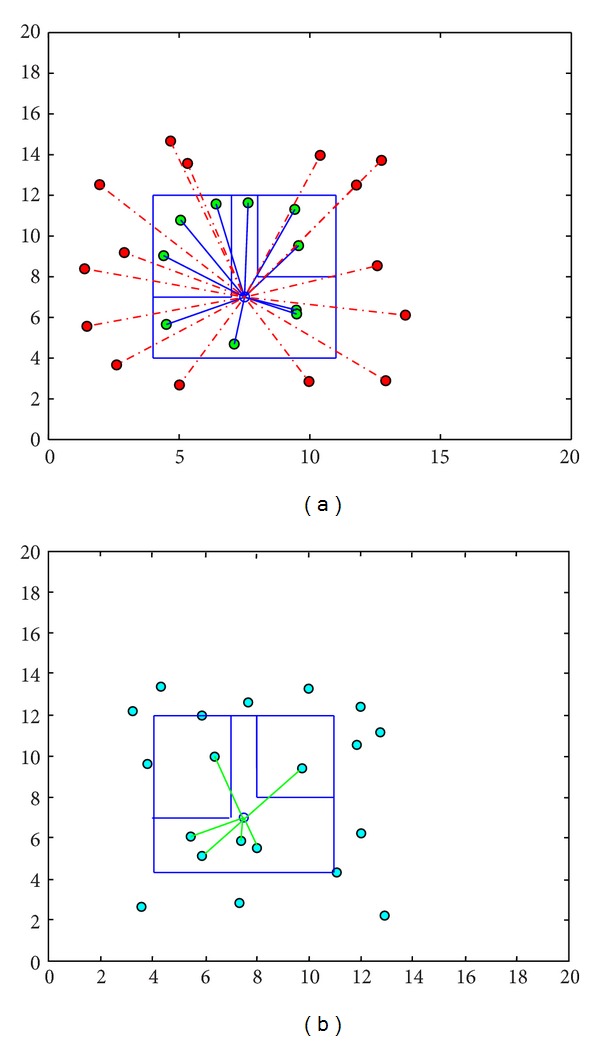
(a) Training and (b) testing of the femtocell device.

Figure 5(a) shows the training stage of the femtocell device. The red dots are the outdoor users and green dots are the indoor users. In Figure 5(b), random users are generated for the femtocell to classify the indoor and outdoor users by using the learning experience. Femtocell only allows connection to the indoor users to be connected. The green connecting lines between the femtocell and the indoor users confirm the proper recognition of the users.

**Figure 6:**
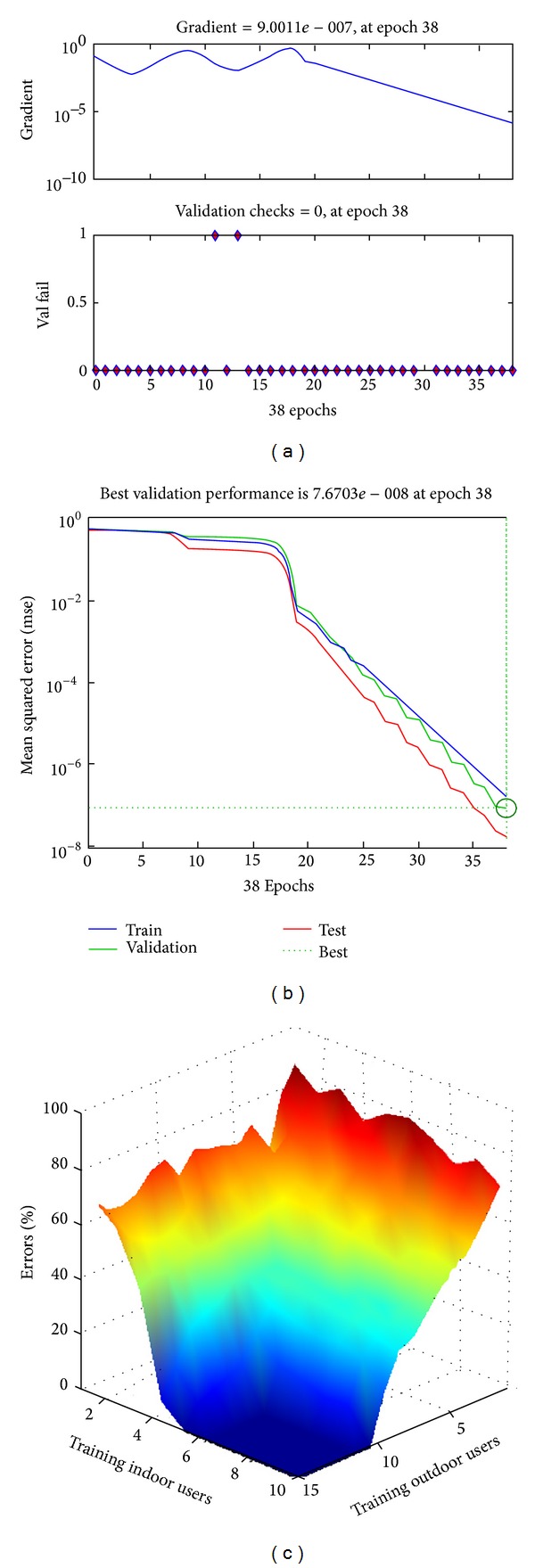
(a) Training state and (b) performance of best validation. (c) Performance of femtocell for different number of samples.

Figures 6(a) and 6(b) show the training state and performance validation state for a simulation with 10 indoor and 15 outdoor training samples. The minimum gradient of the ANN is set to 1 × 10^−6^. In this particular iteration, ANN takes 38 epochs to train up and adjust the values of biases and weights to achieve the minimum gradient value. The “validation graph” shows a downward curve. It confirms that, after every epoch, the latest values of the weights and biases validate the previous training samples.

Figure 6(c) shows the performance of the femtocell in percentage of error for different number of outdoor and indoor training user samples. In every iteration, the network is tested using 20 random users to verify the performance. In both types of users, the error rate is quite high at the beginning. Due to lack of knowledge of the users' behaviour, the system cannot categorize the nature of the randomly created users. For the same number of indoor users, outdoor users' percentage of error rate is higher. This is because of the unpredictable nature of wireless signal propagation from the outdoor users end. The outdoor walls, their shapes, and constructing materials also add more variations in the outdoor users signal strength due to absorption losses and diffraction loss. As a result, the ANN requires higher number of outdoor users training samples for categorizing the users. However, after 5 indoor and 10 outdoor user samples, the network reaches the perfection with error-free user detection. It shows that the performance of the indoor sample is better than the outdoor sample. In the indoor situation, the variation of the signal strength is limited to a certain bound. The effects of indoor free-space loss, refraction, diffraction, reflection, aperture-medium coupling loss, and absorption are comparatively smaller which allows the system to verify any random users signal strength within a certain variation of received power strength. Nevertheless, the number of the sample users always depends on the geographical shape of the houses. The system requires higher number of indoor samples when the variation bounds overlap with the outdoor users variation bound. Such a case is studied below.

**Figure 7:**
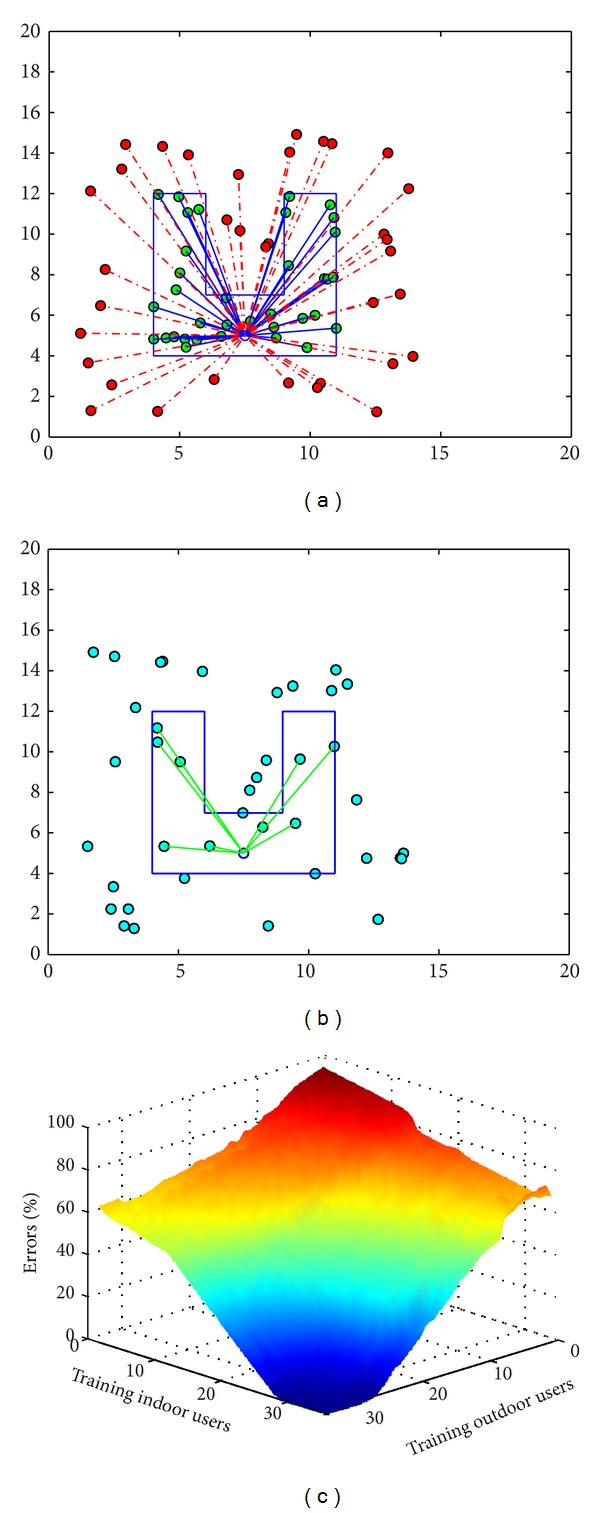
(a) Training and (b) testing process of the femtocell with “U” shaped house. (c) Performance of femtocell for different number of samples.

The proposed method is now tested in a more complex scenario. A “U” shaped house layout is designed to test the performance of the system. In this layout, indoor wall is ignored. Figures 7(a) and 7(b) show the training and testing process of the femtocell network. The challenging shape of the house makes the user pattern more improvised than the previous one. In this case, the system requires higher number of indoor and outdoor training user samples to reach an error-free performance.  Figure 7(c) shows the required number of indoor and outdoor users against the percentage of error occurrences in detecting the users' category. Here the required number of users for both categories is above 25 users. The rest of the performance analysis of the process is done using the previous layout of the house.

### 3.2. Femtocell Network Performance with ANN and AP Clustering Algorithm

**Figure 8:**
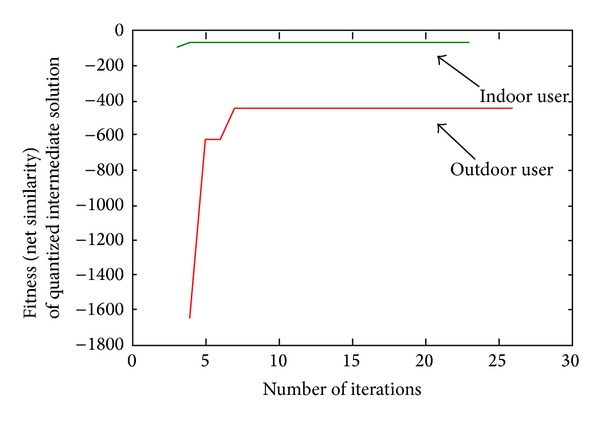
Fitness of AP clustering algorithm for indoor and outdoor users.

AP algorithm clusters the users into subgroups based on their power pattern and selects a representative from each subgroup. Unlike other clustering methods, AP algorithm selects the clusters/subgroups based on the samples nature. If the nature of the sample varies immensely, the number of clusters gets higher. The clustering performance of the AP algorithm is presented in Figure 8 as a form of achieved fitness (net similarities) with respect to the iteration number. Both the outdoor and indoor users reach their best fitness before 8 iterations. However, a safe margin of 25 is kept to ensure the best fitness for both types of users.

**Figure 9:**
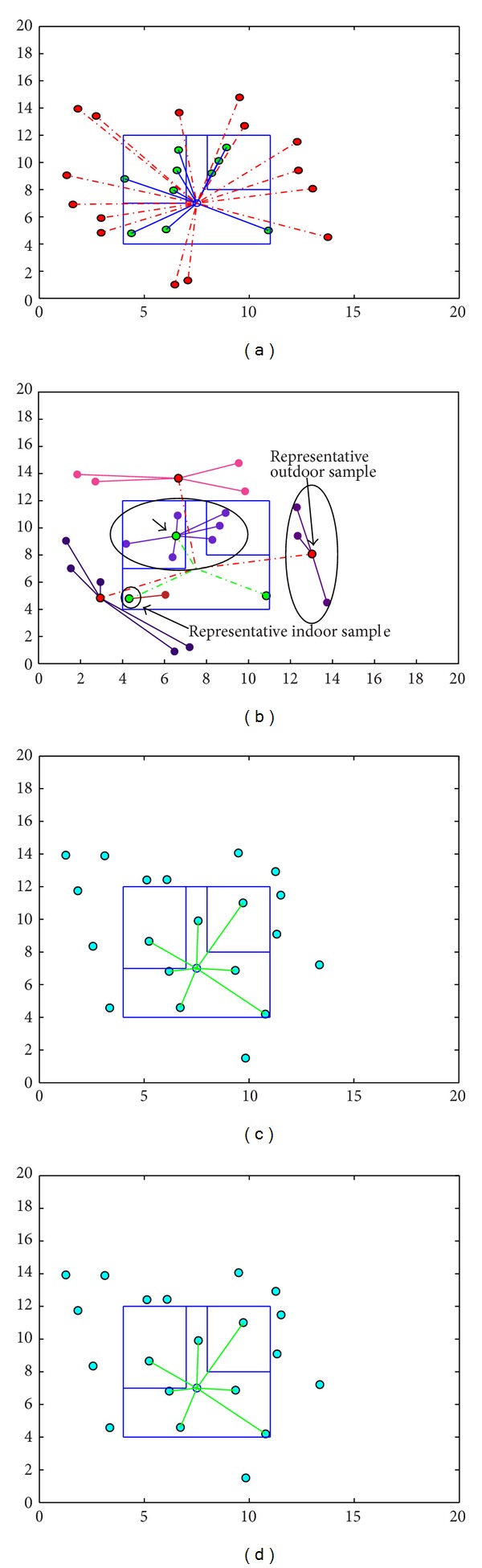
(a) Training with ANN. (b) Training with ANN+AP. (c) Performance of the network with ANN training. (d) Performance of the network with ANN+AP training.

Figure 9(a) shows the general ANN training process. During the training, the ANN adjusts the values of the weights and the biases of the network. In Figure 9(b), the AP algorithm clusters the users based on their similarities: power pattern. A representative has been chosen among the data points of a subgroup, which has most of similarities with the other data points of the subgroup. There might also exist subgroups with only one data point. Figure shows that, instead of training ANN with 15 outdoor users and 10 indoor users, the AP selects 3 outdoor users and 3 indoor users. Figures 9(c) and 9(d) show the performance of the network with and without AP algorithm. For a random simulation, both processes show the same accuracy.

**Figure 10:**
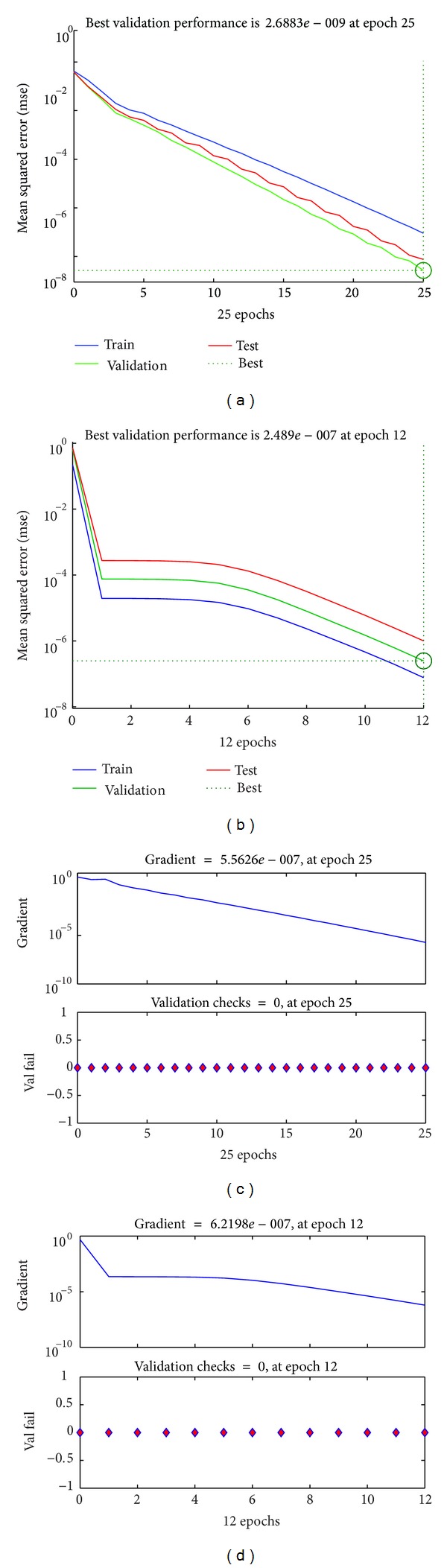
(a) Best validation performance of ANN. (b) Best validation performance of ANN+AP. (c) Training state of ANN. (d) Training state of ANN+AP femtocell network.

Results show that training the ANN in corporation with AP clustering requires less number of training samples. The process takes less number of epochs to reach the gradient's threshold value. For the above simulation, the ANN took 25 epochs while it took 12 epochs using AP clustered samples. The representative of the data points helps the ANN to explore all the possible variations of the characters of the users' power pattern and guide the network to balance the values of weights and the biases with a faster time interval. Figures 10(a) and 10(b) show the mean square rate (MSE) of the training process. Due to higher number of sample data points, the accuracy of the regular ANN training is more precise. However, in the training process with clustered data samples, the mean square error decreases drastically and gets to the desired value with less number of epochs. In Figures 10(c) and 10(d), the validation check shows a good fitness since the number of indoor and outdoor users is chosen from the error-free region achieved in the result in Figure 6(c).

The performance analysis of both processes is shown in Table 2. Randomly, 20 users have been generated every time to test the performance of the network. Each resultant data is an average value of 1000 simulations. The AP+ANN training process takes around 75%–85% less time than the regular ANN training process; meanwhile AP clustering process takes some additional time which makes the total AP+ANN time around 50%–60% less than ANN regular training time. After AP algorithm implementation, the number of epochs also decreases down to 40%. The fraction values of the epochs in Table 2 are expressed by the nearest integer value.

### 3.3. AP Clustering Algorithm versus* K*-Means Clustering Algorithm and Fuzzy* c*-Means Clustering

To justify the selection of AP clustering algorithm over the traditional clustering algorithm, two popular algorithms,* K*-means and fuzzy* c*-means clustering, are compared with AP clustering in the ANN training process.

*K-Means*.* K*-means is one of the simplest unsupervised learning algorithms that solves the well-known clustering problems. It partitions the data set into *k* mutually exclusive clusters and returns the index of the cluster to which it has assigned each observation. Unlike AP,* K*-means creates a single level of clusters and needs the number of clusters assigned before the execution. The algorithm breaks the data set into *k* different clusters. If it is unable to find *k* clusters, it breaks the data set into *k* − 1 clusters. Initially it takes *k* number of random observation data set, which is considered the seeds of the algorithm. Then, it assigns all the other observations to *k* seeds based on their proximity to the seeds. In general sense, the algorithm takes a set of objects *S* and an integer *k* and gives a partition of *S* into subsets *S*_1_,…, *S*_*k*_ defined by *k* cluster centroid locations or centres [36].

*Fuzzy c-Means*. The central idea in fuzzy clustering is the nonunique partitioning of the data in a collection of clusters. Like* K*-means, fuzzy* c*-means creates a single level of clusters and needs the number of clusters assigned before the execution. Cluster centres are randomly initialized and data point (*x*_*i*_) assigned into clusters (*C*_*j*_,  *j* = 1  to  *k*). Distance metric (Euclidean distance are widely used) calculate how far away a point is from a cluster centre. When all data points have been assigned to clusters, new cluster centres (centroids) are calculated. The process of calculating cluster memberships and recalculating cluster centres continues until the cluster centres no longer change from one cycle to the next [37, 38].

**Figure 11:**
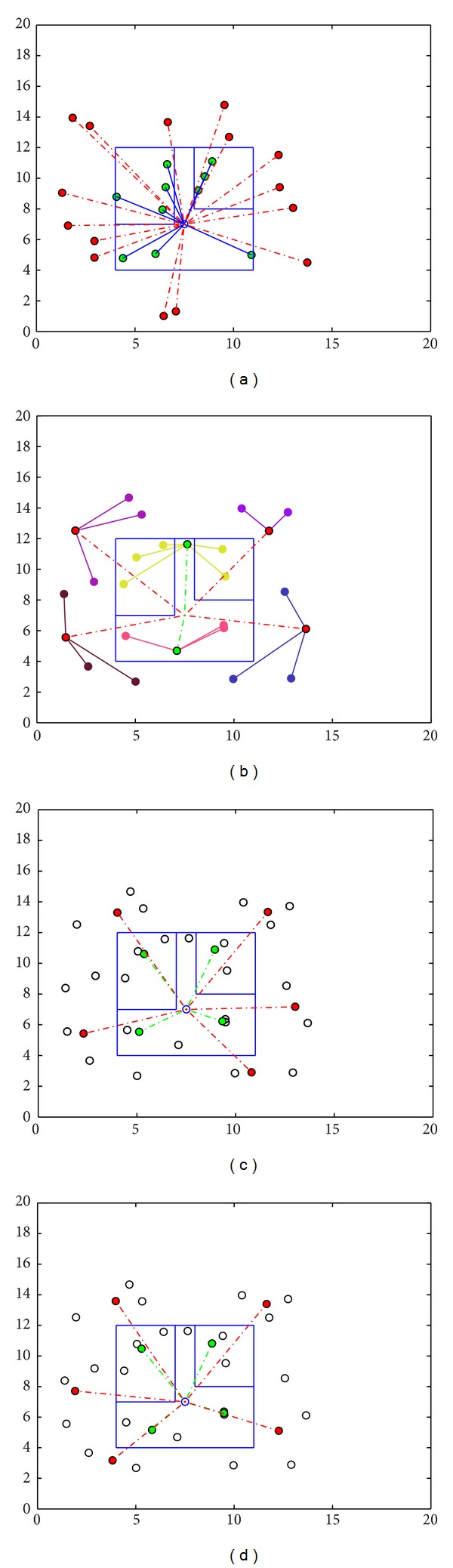
(a) Position of the sample indoor and outdoor users. (b) AP clustering of indoor and outdoor users based on signal strength. (c)* K*-means clustering with 9 clusters. (d) Fuzzy* c*-means clustering with 9 clusters.

Figures 11(b), 11(c), and 11(d) illustrate the representative selection process of AP,* K*-means, and fuzzy* c*-means clustering algorithm in the functioning area. The green dots show the indoor representative points of the data set while the red dots represent the outdoor. In both* K*-means and fuzzy* c*-means, the centroid points are not user data sample; it is a point of each cluster that has a minimum value distance from each of the members of the clusters. In the case of* K*-means, it just executes the distance calculation, whereas fuzzy c-means needs to do a full inverse-distance weighting. To obtain the error-free performance in the ANN,* K*-means and fuzzy* c*-means require different number of clusters each time. A little comparison of the performance is shown in Table 3.

*K*-means minimizes the sum of distances from each data points to its cluster centroid. The process repeats until the sum of distances cannot be decreased further. This process takes more time than AP. On the other hand,* K*-means needs to do a distance calculation, whereas fuzzy* c*-means needs to do a full inverse-distance weighting. Fuzzy* c*-means thus performs slower than both clustering algorithms in this particular case. However, for higher number of data samples, the time increment is a little less than the AP clustering algorithm. Although the overall clustering time of AP algorithm is always less by a fair distance, the number of clusters has to be determined maintaining the same accuracy of the ANN output. Except AP algorithm, the challenge in the other clustering processes mostly lies in selecting the number of clusters to perform an error-free training. On this note, AP algorithm is the best candidate in this process as it selects the number of clusters by itself analysing the samples in every simulation.

## 4. Conclusion

This paper proposed a novel technique to classify the users in closed access femtocell network by using ANN and AP clustering algorithm. The technique is developed using a multielement antenna femtocell device. The power pattern of each user is used to distinguish different level of users. A machine learning process is adopted by using ANN to inaugurate the user recognition feature in the femtocell. After using a certain number of user samples, the femtocell successfully recognizes the indoor and outdoor users. In the later part, AP clustering algorithm is included along with ANN to speed up the training process. Performance analysis shows that the femtocell takes less time to recognize user without compromising the accuracy. Finally, a comparison of AP clustering,* K*-means clustering, and fuzzy* c*-means is showed in the user classification process to justify the selection of AP clustering method. The result shows for same simulation that both* K*-means and fuzzy* c*-means consume more time and give less efficiency.

## Figures and Tables

**Table 1:** System parameters.

System parameters	Value/range
Frequency	2.53 GHz
Number of training indoor users	10
Number of training outdoor users	15
Number of randomly placed users (after training)	20
Femtocell antenna height	1 m
User equipment height	1 m
Frequency	2.6 GHz
UE transmit power (fixed)	13 dBm
Indoor wall loss	5 dB
Outdoor wall loss	10 dB
Shadow fading std.	6 dB
White noise power density	−174 dBm/Hz
Number of neurons in hidden layer	10

**Table 2:** 

Number of samples		ANN training performance		AP + ANN training performance			Performance comparison after AP	
Indoor	Outdoor	Training time (sec.)	Number of epochs∗	AP clustering time (sec.)	Total training time (sec.)	Number of epochs∗	Training time decreases (%)	Number of epochs decreases (%)
5	10	1.5165	21	0.2885	0.7647	13	49.57	38.09
6	11	1.5655	21	0.3633	0.8257	14	47.25	33.33
7	12	1.6221	22	0.3921	0.9071	14	44.07	36.36
8	13	1.6416	23	0.4172	0.934	15	43.10	34.78
9	14	1.6443	24	0.4226	0.9504	16	42.20	33.33
10	15	1.6504	26	0.4244	0.9611	16	41.76	38.46
11	16	1.6541	26	0.439	0.9803	17	40.73	34.61
12	17	1.6669	27	0.4461	0.9848	18	40.92	33.33
13	18	1.6709	27	0.4556	0.9951	19	40.44	29.62
14	19	1.6801	28	0.4671	0.9951	19	40.77	32.14
15	20	1.6819	28	0.4702	0.9964	19	40.75	32.14
16	21	1.688	29	0.4704	0.9982	19	40.86	34.48
17	22	1.7322	30	0.4997	1.0017	21	42.17	30
18	23	1.7767	31	0.5073	1.0397	22	41.48	29.03
19	24	1.7966	32	0.5234	1.0695	22	40.47	31.25
20	25	1.7996	34	0.5484	1.0511	22	41.59	35.29

*The fraction values of the epochs are expressed by the nearest integer values.

**Table 3:** 

Number of samples		ANN + AP performance		ANN + *K*-means performance		ANN + fuzzy *c*-means clustering	
Indoor	Outdoor	Number of samples for error-free operation∗	Clustering + training time (sec.)	Number of samples for error-free operation∗	Clustering + training time (sec.)	Number of samples for error-free operation∗	Clustering + training time (sec.)
5	10	6	0.7647	8	1.2516	8	1.3712
10	15	6	0.9611	9	1.3354	8	1.4157
15	20	7	0.9964	9	1.3847	9	1.4869
20	25	7	1.0511	9	1.4964	9	1.5738

*The fraction values of the epochs are expressed by the nearest integer values.
